# Intestinal Region-Dependent Impact of NFκB-Nrf Crosstalk in Myenteric Neurons and Adjacent Muscle Cells in Type 1 Diabetic Rats

**DOI:** 10.3390/biomedicines12102347

**Published:** 2024-10-15

**Authors:** Bence Pál Barta, Benita Onhausz, Abigél Egyed-Kolumbán, Afnan AL Doghmi, János Balázs, Zita Szalai, Ágnes Ferencz, Edit Hermesz, Mária Bagyánszki, Nikolett Bódi

**Affiliations:** 1 Department of Physiology, Anatomy and Neuroscience, Faculty of Science and Informatics, University of Szeged, 6726 Szeged, Hungary; barta.bence@bio.u-szeged.hu (B.P.B.); onhausz.benita@bio.u-szeged.hu (B.O.); egyed.abigel@bio.u-szeged.hu (A.E.-K.); aldoghmi.afnan@bio.u-szeged.hu (A.A.D.); jbalazs@bio.u-szeged.hu (J.B.); zszalai@bio.u-szeged.hu (Z.S.); bmarcsi@bio.u-szeged.hu (M.B.); 2 Department of Biochemistry and Molecular Biology, Faculty of Science and Informatics, University of Szeged, 6726 Szeged, Hungary; agnes.ferencz@bio.u-szeged.hu (Á.F.); hermesz@bio.u-szeged.hu (E.H.)

**Keywords:** NFκB, Nrf2, myenteric plexus, enteric neurons, intestinal smooth muscle, hyperglycaemia, type 1 diabetes, insulin, gut segments, animal model

## Abstract

Background/Objectives: Type 1 diabetes affects cytokines as potential inducers of NFκB signalling involved in inflammation and neuronal survival. Our goal was to assess the expression of NFκB p65 and its negative regulator, Nrf2, in myenteric neurons and adjacent smooth muscle of different gut segments after chronic hyperglycaemia and immediate insulin treatment. Methods: After ten weeks of hyperglycaemia, intestinal samples of control, streptozotocin-induced diabetic and insulin-treated diabetic rats were prepared for fluorescent immunohistochemistry, immunogold electron microscopy, ELISA and qPCR. Results: In the diabetic rats, the proportion of NFκB p65-immunoreactive myenteric neurons decreased significantly in the duodenum and increased in the ileum. The density of NFκB p65-labelling gold particles increased in the ileal but remained unchanged in the duodenal ganglia. Meanwhile, both total and nuclear Nrf2 density increased in the myenteric neurons of the diabetic duodenum. In smooth muscle, NFκB p65 and Nrf2 density increased in the small intestine of diabetic rats. While on the mRNA level, NFκB p65 and Nrf2 were induced, on the protein level, NFκB p65 increased and Nrf2 decreased in muscle/myenteric plexus homogenates. Insulin treatment had protective effects. Conclusions: Our findings reveal a segment-specific NFκB and Nrf expression in myenteric neurons and ganglionic muscular environments, which may contribute to regional neuronal survival and motility disturbances in diabetes.

## 1. Introduction

Type 1 diabetes is an autoimmune metabolic disorder characterized by the destruction of insulin-producing pancreatic ß-cells. Based on WHO data, both the disease prevalence and its mortality rates are rising rapidly [1]. Diabetes causes multiple organ failure, including cardiomyopathy, retinopathy, nephropathy or neuropathy [2,3,4,5]. Among them, several cardiovascular complications are outstanding risk factors of diabetic deaths. Patients with diabetes often suffer from different gastrointestinal motility disturbances, like gastroparesis, diarrhoea or constipation related to enteric neuropathy. Diabetic damage of enteric neurons is strictly region-dependent and may result in alterations of neurochemical character or even extensive cell loss [6]. Intestinal microbial composition, oxidative circumstances and defence mechanisms basically define the environment of neurons from segment to segment and undergo regional alterations in diabetes [7]. These shifts in oxidant/antioxidant balance promote inflammatory processes that affect enteric neurons.

Cytokines, like tumour necrosis factor alpha (TNFα), and different interleukins (ILs) have a great impact on the regulation of intestinal homeostasis [8,9,10], and contribute to the modulation of enteric neuroinflammation [11,12]. Among others, these cytokines are inducers of nuclear factor kappa-light-chain-enhancer of activated B cell (NFκB) signalling pathways [13,14,15].

The NFκB transcription factor family plays an essential role in mediating rapid cellular responses to different stimuli, and is therefore a key component in immune responses, inflammation and cell survival [16,17]. The NFκB family includes five potential subunits: RelA (p65), RelB and c-Rel, which contain a transactivation domain required for gene transcription, and p50 and p52, which do not [17,18]. These subunits are able to assemble into hetero- and homodimers, from which the p65/p50 dimer appears to be the most abundant [19]. The dimers are located in the cytoplasm in an inactive state. Activation of the NFκB complex via canonical or non-canonical pathways results in NFκB nuclear translocation and regulation of specific gene transcription [20].

NFκB activation is a key point early in the pathogenesis of type 1 diabetes, and it can promote both pro-apoptotic and anti-apoptotic cascades [21]. Growing evidence supports that the cytokine-induced activation of the NFκB pathway contributes to the apoptosis of pancreatic ß cells [22,23]. However, other studies have pointed out its protective role; the NFκB blockade during pancreas development reduced the number of endocrine cells and insulin secretion-related gene expression in the adult pancreas [24]. The NFκB signalling pathway also plays a pivotal role in diabetic complications accompanied by extensive neuronal injuries, among others [21]. The dual function of NFκB regulating both pro- and anti-apoptotic gene expression may result in cell death or promote neuronal survival [25,26,27]. Upregulated NFκB activation caused by advanced glycation end products was observed in peripheral nerves that contribute to diabetic polyneuropathy [21], while the suppression of NFκB alleviates the related symptoms in patients with diabetes [28,29]. On the other hand, it has also been evidenced that NFκB activation is essential to the survival-promoting effect of TNFα on neurons [13,25].

It is well established that there is a molecular and functional crosstalk between the NFκB and nuclear factor erythroid 2-related factor 2 (Nrf2) pathways in the regulation of cellular responses to oxidative stress and inflammation [16,30]. The Nrf2 transcription factor contributes to the anti-inflammatory process through mediating the transcription of different antioxidant proteins [31]. Both NFκB and Nrf2 compete for the same transcriptional co-activator complex; therefore, Nrf2 is able to inhibit the NFκB pathway and serves as a negative regulator [16,30]. Based on that, the NFκB-Nrf2 interplay is a critical component in maintaining the epithelial barrier function and mitigating inflammation in the gastrointestinal tract in response to oxidative injury [32]. Interactions between these two factors fundamentally define inflammatory processes both in neurons and in their environment and influence the severity of diabetic neuropathy [33]. Comprehensive research of different cytokines has been implemented along the gastrointestinal tract, which highlights their roles in diabetes-related segment-specific enteric neuropathy [34,35,36]. However, the expression of NFκB and Nrf2 in enteric ganglia have not been investigated in diabetes.

Therefore, the main aim of this study was to investigate the NFκB and Nrf2 expression in the myenteric ganglia and its muscular environment in different gut segments of diabetic rats with untreated chronic hyperglycaemia and insulin treatment.

## 2. Materials and Methods

### 2.1. Animal Model

Wistar rats (200–300 g, male, Toxi-Coop Zrt., Balatonfüred, Hungary) were randomized into STZ-induced diabetic (diabetic; *n* = 14), insulin-treated STZ-induced diabetic (insulin-treated diabetic; *n* = 13) and age- and sex-matched control (*n* = 14) groups. Hyperglycaemia was induced by a single injection of STZ (60 mg/kg, i.p., S0130, Sigma–Aldrich, Budapest, Hungary) [6,34,37]. The rats were considered diabetic if their blood glucose level was above 18 mmol/L. The insulin-treated group received injections of insulin (Humulin M3, HI0719, Eli Lilly Nederland, Utrecht, The Netherlands) twice a day (3-3 IU, s. c.) during the 10-week experiment. Equivalent volumes of saline were injected (s. c.) in the diabetics and the controls. Monitoring of the glycaemic state and weight of the animals, and reasons for animal exclusion from this study, were detailed previously [35,38]. The principles of the National Institutes of Health (Bethesda, MD, USA) guidelines and the EU directive 2010/63/EU were followed, and our research was approved by the National Scientific Ethical Committee on Animal Experimentation (National Competent Authority).

### 2.2. Tissue Handling

Ten weeks after the induction of hyperglycaemia, the rats were killed [35,38] according to animal experiment license XX./1636/2019. The intestinal tissues of experimental groups were dissected and rinsed in 0.05 M phosphate buffer (PB; pH 7.4). Intestinal samples were taken from the duodenum, the ileum and the colon [35,36], and processed for quantitative PCR, ELISA, double-labelling fluorescent immunohistochemistry and quantitative electron microscopy. For the qPCR and ELISA, the samples were cut along the mesentery, the layers of mucosa and submucosa were removed and intestinal smooth muscle layers, including the myenteric plexus (MUSCLE-MP), were snap-frozen in liquid nitrogen and stored at −80 °C until use. For fluorescent immunohistochemistry, the gut samples were cut along the mesentery, pinched flat and fixed overnight (4 °C) in 4% formaldehyde solution (37308, Molar-Chemicals Kft., Halásztelek, Hungary). After washing, the mucosa, submucosa and circular smooth muscle were removed, and whole-mount preparations containing the myenteric plexus and the longitudinal smooth muscle were prepared. For the post-embedding immunogold technique, small pieces of different segments were fixed in 2% paraformaldehyde—2% glutaraldehyde solution and in 2% OsO_4_. After rinsing in buffer and dehydrating in increasing ethanol concentrations and acetone, they were embedded in Embed812 (14900, Electron Microscopy Sciences, Hatfield, PA, USA).

### 2.3. RNA Extraction, Reverse Transcription and Quantitative PCR

Frozen fragments were pulverized under liquid nitrogen, homogenized in RNA bee reagent (CS-104B, Tel-Test Inc., Friendswood, TX, USA) and total RNA was prepared according to the manufacturer’s recommendations. RNA purity and concentration was measured with a NanoDrop ND-1000 spectrophotometer (Thermo Scientific, Waltham, MA, USA). The concentration of the RNA was calculated using the A260 = 1.0 equivalent to ~40 µg/mL single-stranded RNA equation. First-strand cDNAs were synthesized using 5 µg total RNA and the Maxima H Minus First Strand cDNA Synthesis Kit with oligo(dT)18 priming, according to the manufacturer (Thermo Scientific, Waltham, MA, USA: K1652). qPCR was applied in Applied Biosystems 7500 Fast Real-Time system using Luminaris Color HiGreen qPCR Master Mix, Low ROX (Thermo Scientific, Waltham, MA, USA: K0371). The qPCR reactions were performed in 96-well plates containing 20 µL reaction mix/well with a temperature program of 95 °C for 10 min (initial denaturing), then 45 cycle of 15 s at 95 °C and 40 s at 63 °C. The RT-qPCR reactions for each sample were performed in triplicate to increase the reliability of the measurements. The Ct values of all samples were normalized to the internal control (18S rRNA) and gene expression was calculated in terms of the 2^−ΔΔCt^ method [39].

### 2.4. Primers

Data of the primers (Bio Basic Canada Inc., Markham, ON, Canada) are summarized in Table 1.

### 2.5. Measurement of Tissue NFκB and Nrf2 Concentrations

MUSCLE-MP samples were fresh-frozen in liquid nitrogen, crushed into powder and homogenized in 500 µL homogenizing buffer (100 µL protease inhibitor cocktail (Sigma-Aldrich, Budapest, Hungary) in 20 mL 0.05 M PB). Homogenates were centrifuged at 5000 rpm for 20 min at 4 °C. The NFκB p65 and Nrf2 levels of the homogenates were determined by means of quantitative ELISA according to the manufacturer’s instructions (NFκB p65: GA-E4022RT; Nrf2: GA-E4033RT; GenAsia Biotech Co., Ltd., Shanghai, China). Optical density was measured at 450 nm (Benchmark Microplate Reader; Bio-Rad, Budapest, Hungary). Tissue NFκB p65 and Nrf2 concentrations were expressed as ng/mg protein.

### 2.6. Bradford Protein Micromethod

To determine the total protein content of intestinal samples, a commercial protein assay kit was used. Bradford reagent was mixed with the samples and, after 10 min incubation, the samples were assayed by a spectrophotometer (595 nm). The level of total protein content was measured as mg protein/mL.

### 2.7. Fluorescent Immunohistochemistry

Whole mounts derived from different segments of small intestine were immunostained with NFκB p65 and HuC/HuD pan-neuronal marker or triple-labelled with NFκB p65, Nrf2 and Peripherin pan-neuronal marker. After blocking [35,38], the whole mounts were incubated overnight with anti-NFκB p65 (F-6) (mouse monoclonal IgG; 1:50; Cat.No.sc-8008, Santa Cruz Biotechnology, Dallas, TX, USA) and anti-HuC/HuD (rabbit monoclonal IgG; 1:200; Cat.No.ab184267, Abcam, Cambridge, UK) or with anti-NFκB p65 (F-6) (mouse monoclonal IgG; 1:50; Cat.No.sc-8008, Santa Cruz Biotechnology, Dallas, TX, USA), anti-Nrf2 (rabbit polyclonal, 1:100; Cat.No.ab31163, Abcam, Cambridge, UK) and anti-Peripherin 1 (guinea pig polyclonal IgG; 1:500; Cat.No.424 004, Synaptic System, Goettingen, Germany) primary antibodies (4 °C). After washing in TBS with 0.025% Triton X-100 (A16046, Molar-Chemicals Kft., Halásztelek, Hungary), sections were incubated with anti-mouse Cy3 (1:200; Cat.No.115-165-003, Jackson ImmunoResearch Laboratories, West Grove, PA, USA), anti-rabbit Alexa Fluor 488 (1:200; Cat.No.A11008, Invitrogen, Thermo Fisher Scientific, Waltham, MA, USA) and anti-guinea pig Cy5 (1:200; Cat.No.ab102372, Abcam, Cambridge, UK, in the case of triple-labelling) secondary antibodies for 1 h at room temperature. No immunoreactivity was detected on negative controls omitting the primary antibodies. Whole mounts were mounted and photographed with a Zeiss Imager Z.2 fluorescent microscope (Axiocam 506 mono camera) (Zeiss, Jena, Germany). At least one thousand myenteric neurons were taken from the duodenum and the ileum (per group), and the proportion of NFκB p65-immunoreactive myenteric neurons were counted per ganglia.

### 2.8. Quantitative Post-Embedding Immunohistochemistry

From embedded blocks of small gut segments of each experimental group, ultrathin (70 nm) sections were prepared and mounted on nickel grids for NFκB p65 and Nrf2 immunogold labelling. These sections (3 grids/block) were incubated overnight in anti-NFκB p65 (F-6) (mouse monoclonal IgG; 1:25; Cat.No.sc-8008, Santa Cruz Biotechnology, Dallas, TX, USA) or anti-Nrf2 (rabbit polyclonal; 1:25; Cat.No.ab31163, Abcam, Cambridge, UK) primary antibodies, followed by anti-mouse IgG (conjugated to 18 nm colloidal gold particles; 1:20; Cat.No.115-215-071, Jackson ImmunoResearch, West Grove, PA, USA; or conjugated to 10 nm gold particles; 1:30; Cat.No.G7652, Sigma-Aldrich, Louis, MO, USA) or anti-rabbit IgG (conjugated to 18 nm gold particles; 1:20; Cat.No.111-215-144, Jackson ImmunoResearch, West Grove, PA, USA) secondary antibodies for 3 h. No immunoreactivity was detected on negative controls omitting the primary antibodies. After counterstaining [40], the sections were photographed with a JEOL JEM 1400 transmission electron microscope. The subcellular localization and quantitative features of the gold particles’ labelling NFκB p65 or Nrf2 were determined in the myenteric ganglia and surrounding smooth muscle cells. Fifty digital photographs of ten myenteric ganglia and thirty digital photographs of intestinal smooth muscle cells/gut segments/conditions were made at a magnification of 20,000× with a transmission electron microscope (JEOL JEM 1400, JEOL, Tokyo, Japan) and the TEM Center 1.6.9 software (JEOL, Tokyo, Japan). The intensity of the labelling was expressed as the total number of gold particles per unit area (µm^2^).

### 2.9. Statistical Analysis

A Kruskal–Wallis test and a Dunn’s multiple comparisons test were used for statistical analysis (GraphPad Prism 6.0; GraphPad Software Inc, San Diego, CA, USA). A probability of *p* < 0.05 was set as the level of significance. Data were expressed as mean ± SEM.

## 3. Results

### 3.1. Disease Characteristics of Type 1 Diabetic Rats

During the 10 weeks of the experiment, the weight and glycaemic features of the experimental animals were monitored (Table 2). Diabetic rats were characterised by a long-lasting chronic hyperglycaemia with a 29.46 ± 0.87 mmol/L average blood glucose concentration, which was almost five times higher than that of the control animals (5.92 ± 0.11 mmol/L; *p* < 0.0001). Immediate insulin treatment prevented extremely high glucose levels; however, the values were still higher than in the controls (12.98 ± 1.5 mmol/L; *p* < 0.05). All the animals gained weight during the experiment, but the final body weight of diabetic rats was notably lower compared with the insulin-treated diabetics and controls.

### 3.2. Diabetic Alterations of Intestinal NFκB Expression

#### 3.2.1. Quantitative Changes in NFκB p65 mRNA Expression

**Figure 1:**
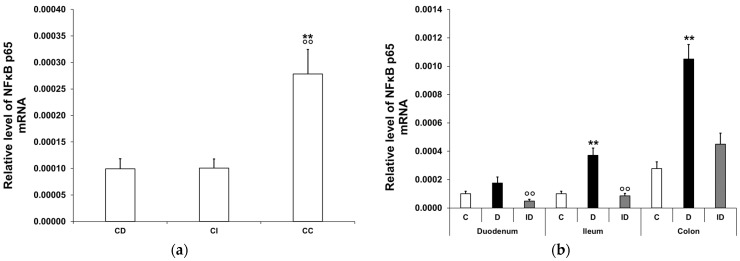
Relative levels of NFκB p65 mRNA. (**a**) Relative levels of NFκB p65 mRNA in tissue homogenates from different gut segments of control rats. The expression level of NFκB p65 mRNA was nearly three times higher in the colon than in the small intestinal segments of controls. Data were expressed as means ± SEM. ** *p* < 0.01 (compare with CD); ^oo^ *p* < 0.01 (between CI and CC). CD—control duodenum, CI—control ileum, CC—control colon. (**b**) Effects of long-lasting hyperglycaemia and insulin treatment on the relative level of NFκB p65 mRNA in tissue homogenates from different gut segments. In the diabetics, the relative level of NFκB p65 mRNA displayed a more than 3-fold increase in the tissue homogenates of the colon and ileum, which was prevented by insulin treatment. Data were expressed as means ± SEM. ** *p* < 0.01 (compare to C); ^oo^ *p* < 0.01 (between D and ID). C—controls, D—diabetics, ID—insulin-treated diabetics.

In the controls, the expression level of NFκB p65 mRNA was highest in the colon, while a third of this value was measured in the duodenum (*p* < 0.01) and the ileum (*p* < 0.01) (Figure 1a). In diabetics, the relative level of NFκB p65 mRNA increased regionally along the intestinal tract. It was more than 3.5-fold higher in tissue homogenates of both the colon (*p* < 0.01) and the ileum (*p* < 0.01) of diabetics relative to the control group, while no significant changes were observed in the diabetic duodenum (Figure 1b). In the insulin-treated diabetic group, the NFκB p65 mRNA levels were close to the control values in all investigated gut segments (Figure 1b).

#### 3.2.2. NFκB p65 Protein Level in Smooth Muscle/Myenteric Plexus Homogenates

**Figure 2:**
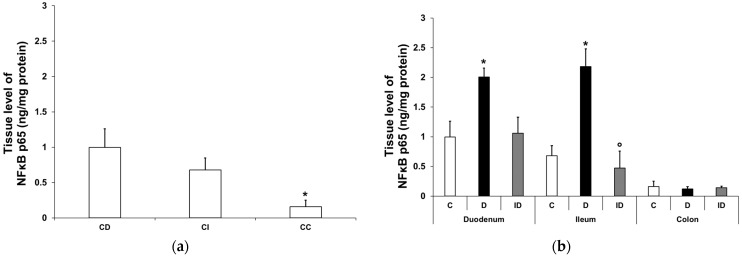
Tissue levels of NFκB p65. (**a**) Tissue levels of NFκB p65 in intestinal smooth muscle/myenteric plexus homogenates from different gut segments of control rats. The tissue level of NFκB p65 displayed a significant decrease from the duodenum to the colon of controls. Data were expressed as means ± SEM. * *p* < 0.05 (compare with CD). CD—control duodenum, CI—control ileum, CC—control colon. (**b**) Effects of long-lasting hyperglycaemia and insulin treatment on the tissue levels of NFκB p65 in smooth muscle/myenteric plexus homogenates from different gut segments. In the diabetic rats, the NFκB p65 level was doubled in the duodenum and tripled in the ileum, while it did not change in the colon. Immediate insulin treatment was completely protective against diabetes-related changes. Data were expressed as means ± SEM. * *p* < 0.05 (compare with C); ^o^ *p* < 0.05 (between D and ID). C—controls, D—diabetics, ID—insulin-treated diabetics.

In the control conditions, the tissue level of NFκB p65 displayed a significant decrease from the duodenum to the colon (0.997 ± 0.27 vs. 0.16 ± 0.09 ng/mg; *p* < 0.05; Figure 2a). In the diabetic muscle/myenteric plexus homogenates, the NFκB p65 level increased markedly in the small intestine compared with the control samples; it doubled in the duodenum (2.01 ± 0.15 vs. 0.997 ± 0.27 ng/mg; *p* < 0.05) and tripled in the ileum (2.18 ± 0.3 vs. 0.68 ± 0.17 ng/mg; *p* < 0.05), which was completely prevented by insulin treatment (Figure 2b). In colonic homogenates, neither the diabetic nor the insulin-treated diabetic samples showed any changes in their NFκB p65 level in our 10-week diabetic rat model (Figure 2b).

#### 3.2.3. Proportion of NFκB p65-Immunoreactive Myenteric Neurons

**Figure 3:**
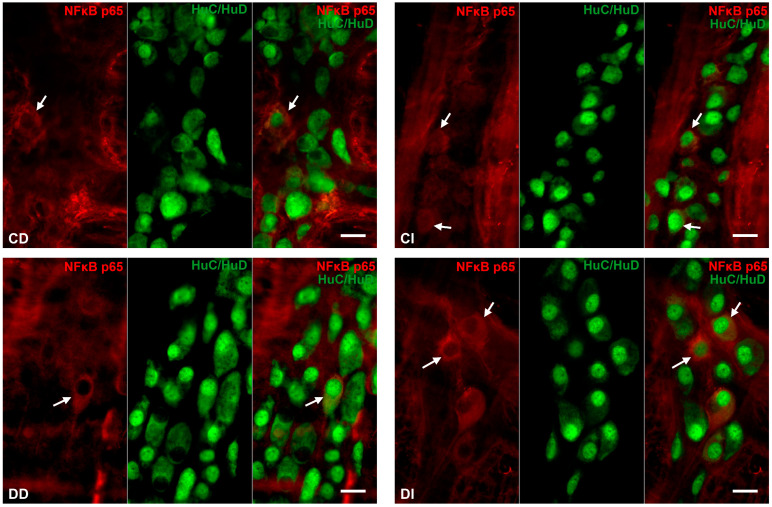

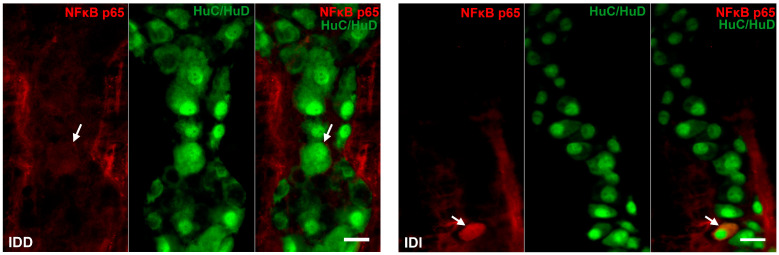
Representative fluorescent micrographs of whole-mount preparations of myenteric ganglia from the duodenum and ileum of control, diabetic and insulin-treated diabetic rats after NFκB p65-HuC/HuD double-labelling immunohistochemistry. HuC/HuD as a pan-neuronal marker was applied to label myenteric neurons. CD—control duodenum, CI—control ileum, DD—diabetic duodenum, DI—diabetic ileum, IDD—insulin-treated diabetic duodenum, IDI—insulin-treated diabetic ileum, arrows—NFκB p65-immunoreactive myenteric neurons. Scale bars: 20 μm.

**Figure 4:**
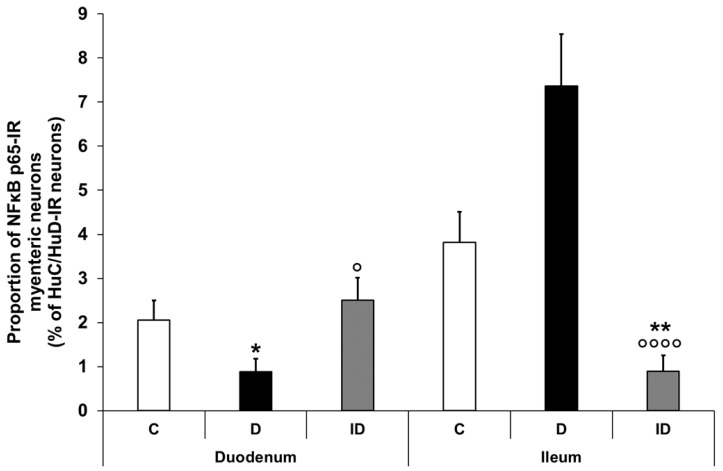
Proportion of NFκB p65-immunoreactive myenteric neurons in the duodenum and ileum of control, diabetic and insulin-treated diabetic rats. In the diabetics, the proportion of NFκB p65-immunoreactive myenteric neurons was significantly decreased in the duodenum and increased in the ileum, which was prevented by immediate insulin treatment. Data were expressed as mean ± SEM. * *p* < 0.05, ** *p* < 0.01 (compare with C); ^o^ *p* < 0.05, ^oooo^ *p* < 0.0001 (between D and ID). C—controls, D—diabetics, ID—insulin-treated diabetics.

Double-labelling immunofluorescence unveiled regional differences in NFκB p65 immunoreactivity in the myenteric ganglia along the small intestine both in the controls and the diabetics. In the neurons, different labelling patterns of NFκB immunoreactivity was observed according to the following types: only cytosolic labelling; intensity of labelling was higher in cytoplasm than nucleus; intensity of labelling was homogenous in cytoplasm and nucleus (Figure 3). The proportion of NFκB p65-IR myenteric neurons compared with the total number of myenteric neurons was 2% in the duodenal, while it was almost double, 3.8%, in the ileal ganglia of control animals (Figure 4). In diabetics, this proportion was significantly decreased in the myenteric ganglia of the duodenum (less than 1%) and was increased in the ileum (more than 7%) relative to the controls (Figure 4). Insulin treatment completely prevented the diabetic changes in the proportion of NFκB p65-IR neurons in the duodenum; however, it decreased that proportion significantly below the control values in the ileum (Figure 4).

#### 3.2.4. Quantitative Distribution of NFκB p65-Labelling Gold Particles in Myenteric Ganglia and Intestinal Smooth Muscle

**Figure 5:**
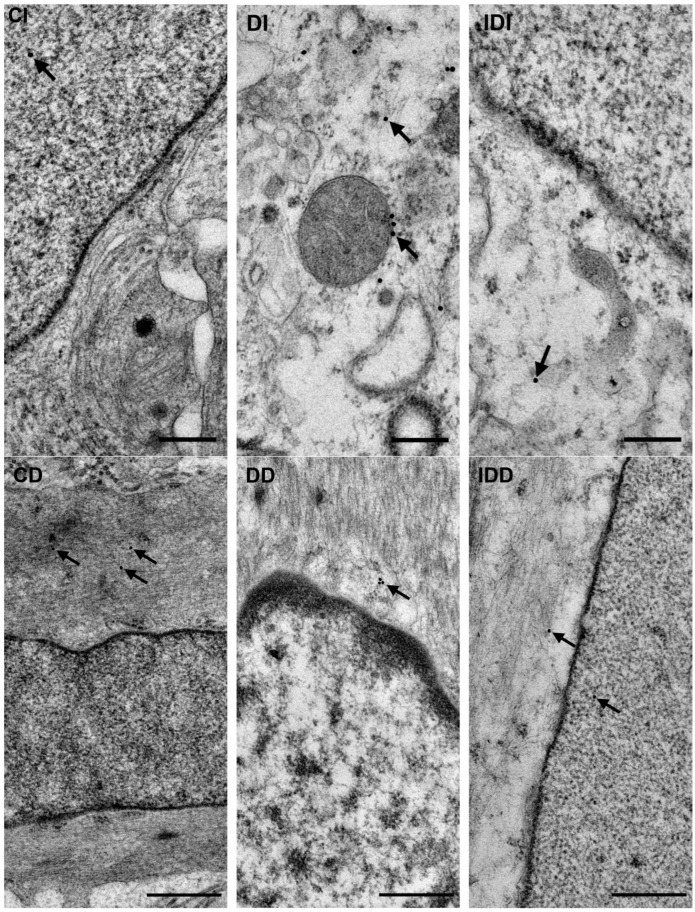
Representative electron micrographs of portions of the perikaryon and nuclei of myenteric neurons from ileum and intestinal smooth muscle cells from duodenum of control, diabetic and insulin-treated diabetic rats after NFκB p65 post-embedding immunohistochemistry. CD—control duodenum, CI—control ileum, DD—diabetic duodenum, DI—diabetic ileum, IDD—insulin-treated diabetic duodenum, IDI—insulin-treated diabetic ileum, arrows—18 nm gold particles’ labelling NFκB p65. Scale bars: 250 nm.

**Figure 6:**
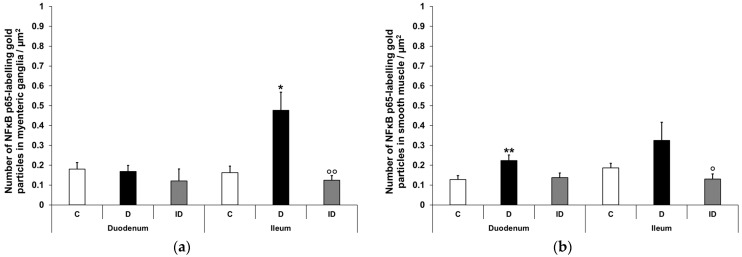
Quantification of gold particles’ labelling NFκB p65 in myenteric ganglia (**a**) and intestinal smooth muscle (**b**) from different gut segments of control, diabetic and insulin-treated diabetic rats. The number of NFκB p65-labelling gold particles increased in the ileal myenteric ganglia and intestinal smooth muscle of both the duodenum and the ileum of diabetic animals relative to the controls, which was prevented by insulin. Data were expressed as means ± SEM. * *p* < 0.05, ** *p* < 0.01 (compare with C); ^o^ *p* < 0.05, ^oo^ *p* < 0.01 (between D and ID). C—controls, D—diabetics, ID—insulin-treated diabetics.

Quantitative analysis of NFκB p65 expression in the myenteric ganglia was made after post-embedding immunogold electron microscopy using 18 nm gold particles to label NFκB p65 (Figure 5). The total density of gold particles displayed a marked increase in the myenteric ganglia of the diabetic ileum (0.48 ± 0.09 vs. 0.16 ± 0.03; *p* < 0.05), in both the nuclear and the cytoplasmic levels, but it did not change in the diabetic duodenum relative to the controls (0.17 ± 0.03 vs. 0.18 ± 0.03; Figure 6a). In the case of intestinal smooth muscle surrounding the myenteric ganglia, the total density of gold particles enhanced significantly in the duodenum (0.22 ± 0.03 vs. 0.13 ± 0.02; *p* < 0.01) and slightly increased in the ileum (0.32 ± 0.09 vs. 0.19 ± 0.02) of diabetic samples relative to the control group (Figure 6b). Regarding the intracellular distribution, the density of gold particles increased to a similar extent in both the cytoplasm and the nuclei of smooth muscle cells in both the duodenum and the ileum. Insulin treatment prevented all diabetic alterations of NFκB p65 gold particles’ density in the small intestine (Figure 6).

### 3.3. Diabetic Alterations of Intestinal Nrf2 Expression

#### 3.3.1. Quantitative Changes in Nrf2 mRNA Expression

**Figure 7:**
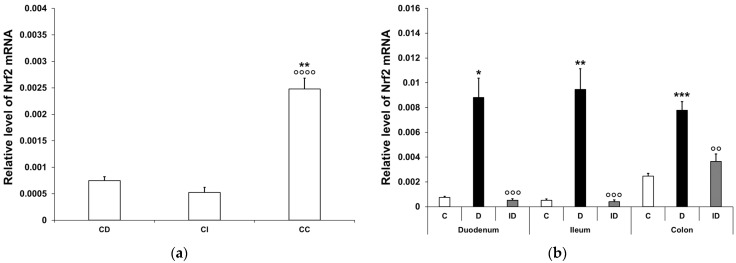
Relative levels of Nrf2 mRNA. (**a**) Relative levels of Nrf2 mRNA in tissue homogenates from different gut segments of control rats. The expression level of Nrf2 mRNA was multiple times higher in the colon than in the small intestinal segments of the controls. Data were expressed as means ± SEM. ** *p* < 0.01 (compare with CD); ^oooo^ *p* < 0.0001 (between CI and CC). CD—control duodenum, CI—control ileum, CC—control colon. (**b**) Effects of long-lasting hyperglycaemia and insulin treatment on the relative level of Nrf2 mRNA in tissue homogenates from different gut segments. In the diabetics, the relative level of Nrf2 mRNA displayed a robust increase in all segments along the duodenum–ileum–colon axis, which was prevented by insulin treatment. Data were expressed as means ± SEM. * *p* < 0.05, ** *p* < 0.01, *** *p* < 0.001 (compare with C); ^oo^ *p* < 0.01, ^ooo^ *p* < 0.001 (between D and ID). C—controls, D—diabetics, ID—insulin-treated diabetics.

In the controls, the Nrf2 mRNA level was similarly low in the duodenum and the ileum, while in the colonic samples, it was multiple times higher (3-fold higher relative to the duodenum (*p* < 0.01) and 4.5-fold higher relative to the ileum) (*p* < 0.0001; Figure 7a). In the diabetic animals, the relative level of Nrf2 mRNA was robustly increased in all segments along the duodenum–ileum–colon axis. The greatest induction was demonstrated in the ileal homogenates of diabetic samples, where the Nrf2 mRNA level was nearly 18-fold higher than that of the control values (*p* < 0.01). Furthermore, an 11-fold induction was revealed in the duodenum (*p* < 0.05) and a 3-fold increase was demonstrated in the colon (*p* < 0.001) of diabetics (Figure 7b). In the insulin-treated diabetic group, the Nrf2 mRNA levels remained close to the control levels in all segments (Figure 7b).

#### 3.3.2. Nrf2 Protein Level in Smooth Muscle/Myenteric Plexus Homogenates

**Figure 8:**
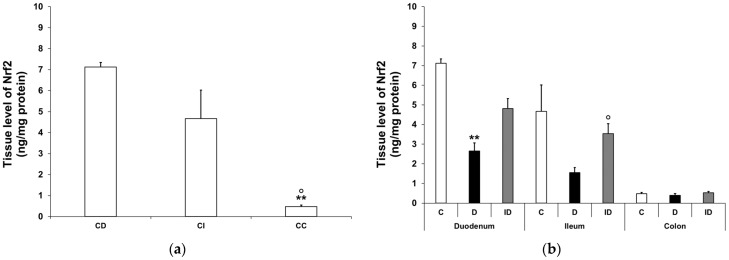
Tissue levels of Nrf2. (**a**) Tissue levels of Nrf2 in intestinal smooth muscle/myenteric plexus homogenates from different gut segments of control rats. The tissue levels of Nrf2 were significantly higher in the small intestine than the colon of the controls. Data were expressed as means ± SEM. ** *p* < 0.01 (compare with CD); ^o^ *p* < 0.05 (between CI and CC). CD—control duodenum, CI—control ileum, CC—control colon. (**b**) Effects of long-lasting hyperglycaemia and insulin treatment on the tissue levels of Nrf2 in smooth muscle/myenteric plexus homogenates from different gut segments. In the diabetic rats, the Nrf2 level was decreased in the duodenum and ileum, while it did not change in the colon. Immediate insulin treatment was protective against diabetes-related changes. Data were expressed as means ± SEM. ** *p* < 0.01 (compare with C); ^o^ *p* < 0.05 (between D and ID). C—controls, D—diabetics, ID—insulin-treated diabetics.

Nrf2 tissue levels exhibited significant differences between small and large intestinal segments, even in the control rats, e.g., it was 15-fold higher in the duodenum than in the colon (7.12 ± 0.22 vs. 0.48 ± 0.07 ng/mg; *p* < 0.01; Figure 8a). In diabetic intestinal homogenates, the level of Nrf2 dropped significantly in the duodenum (2.65 ± 0.42 vs. 7.12 ± 0.22 ng/mg; *p* < 0.01) and displayed a decreasing tendency in the ileum, but remained unchanged in the colon relative to the control levels. A protective effect of immediate insulin treatment in the small intestinal regions was observed at least in part (Figure 8b).

#### 3.3.3. Quantitative Evaluation of Nrf2 Immunohistochemistry in Myenteric Ganglia and Intestinal Smooth Muscle

**Figure 9:**
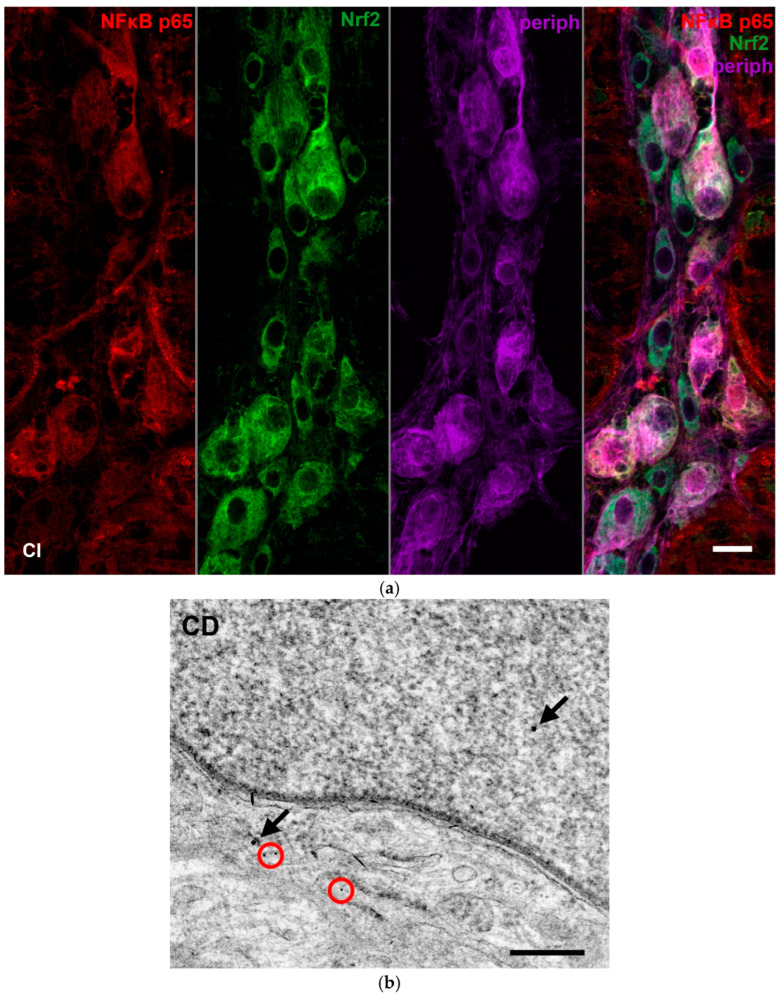
Representative fluorescent micrograph of a whole-mount preparation of myenteric ganglia from the ileum of a control rat after NFκB p65-Nrf2-Peripherin triple-labelling immunohistochemistry (**a**). Pan-neuronal Peripherin was used to label myenteric neurons. Representative electron micrograph of a portion of the perikaryon and nucleus of a myenteric neuron from duodenum of a control rat after NFκB p65-Nrf2 post-embedding immunohistochemistry (**b**). Red circles—10 nm gold particles’ labelling NFκB p65, arrows—18 nm gold particles’ labelling Nrf2. CI—control ileum, CD—control duodenum. Scale bars: 20 µm (**a**), 250 nm (**b**).

Double-labelling fluorescent immunohistochemistry and immunogold electron microscopy have demonstrated NFκB p65 and Nrf2 co-immunoreactivity in enteric neurons on whole-mount preparations and ultrathin sections of myenteric ganglia (Figure 9).

**Figure 10:**
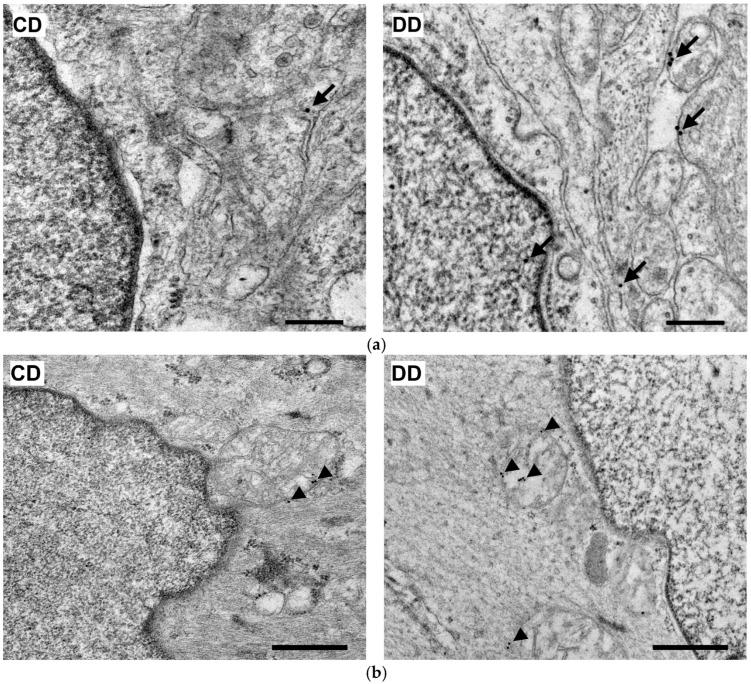
Representative electron micrographs of portions of the perikaryon and nuclei of myenteric neurons (**a**) and intestinal smooth muscle cells (**b**) from the duodenum of control and diabetic rats after Nrf2 post-embedding immunohistochemistry. CD—control duodenum, DD—diabetic duodenum. Arrows—18 nm gold particles’ labelling Nrf2 in myenteric neurons, arrowheads—18 nm gold particles’ labelling Nrf2 in muscle cells. Scale bars: 250 nm.

**Figure 11:**
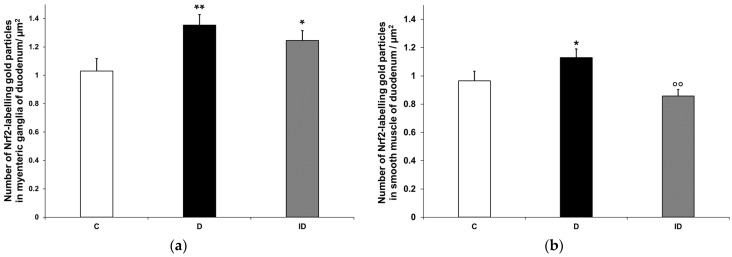
Quantification of gold particles’ labelling Nrf2 in myenteric ganglia (**a**) and intestinal smooth muscle (**b**) from the duodenum of control, diabetic and insulin-treated diabetic rats. The number of Nrf2-labelling gold particles increased in the myenteric ganglia and smooth muscle of diabetic duodenum relative to controls, which was partially prevented by insulin treatment. Data were expressed as means ± SEM. * *p* < 0.05, ** *p* < 0.01 (compare with C); ^oo^ *p* < 0.01 (between D and ID). C—controls, D—diabetics, ID—insulin-treated diabetics.

Quantification of Nrf2 immunogold labelling was completed in both myenteric ganglia and neighbouring smooth muscle cells (Figure 10a,b). Density of gold particles labelling Nrf2 increased significantly in the ganglia (*p* < 0.01) and also in their muscular environment (*p* < 0.05) of diabetic rats compared with controls, which was prevented by insulin treatment (Figure 11a,b).

Moreover, the diabetes-related enhancement of Nrf2 density resulted from the increased number of gold particles in both the neuronal perikarya and nuclei in the case of the myenteric ganglia, while it had a mainly cytoplasmic origin in the muscle cells (Table 3).

## 4. Discussion

The present study provides the first evidence of intestinal region-dependent alterations in both NFκB and Nrf signalling in enteric neurons and their muscular environment of type 1 diabetic rats. Beneficial effects of immediate insulin treatment on NFκB and Nrf expression were also observed in our chronic hyperglycaemic rat model, even if the blood glucose concentrations were not at control levels.

Earlier findings [34,35] regarding the strictly regional expression of different cytokines in distinct gut segments presumably contributed to the regionally induced NFκB expression in the diabetic gut. However, NFκB induction showed opposite trends in mRNA and protein levels along the intestinal axis. While the increase in NFκB p65 mRNA expression was pronounced in distal gut segments of diabetics, the NFκB p65 protein concentration was higher in small intestinal homogenates, with low and unchanged levels in the colon of the diabetic group. In the literature, all variations of NFκB p65 mRNA and protein changes have been demonstrated, e.g., increased mRNA expression was not followed by changes in protein content in human corneal fibroblasts under hypoxic conditions [41]. Unchanged NFκB mRNA levels accompanied by increased protein content [42] or enhancement of NFκB expression at both levels [43] have also been observed in different in vitro and in vivo studies. These reflect the complex regulation of NFκB signalling at multiple levels, including post-translational modifications [44]. The fact that diabetes-related changes in NFκB p65 mRNA and protein levels differed from segment to segment within the gut emphasizes the impact of the regional environmental milieu along the duodenum–ileum–colon axis and certainly contributes to the regional involvement of enteric neurons in diabetic damage.

Based on the protein measurement findings, the proximal and distal part of the small intestine was the focus of our immunofluorescence investigations. In the small intestine of healthy controls, approximately 2–4% of the total myenteric neuronal population displayed immunoreactivity for NFκB p65. In diabetic rats, the proportion of NFκB p65-IR myenteric neurons was significantly rearranged; it halved in the duodenum and doubled in the ileum. These opposite alterations suggest the activation of the NFκB system in the ileum but not in the duodenum. Besides other techniques, immunofluorescence is also appropriate to monitor the cellular states of NFκB activity [45]. The activation of NFκB as a key element of rapid cellular responses can be characterized as a dynamic system, in which different activation states, like constitutive, low and high, exist [46,47]. Among the NFκB-IR neurons, we also observed different patterns of immunolabelling from fully cytosolic to varying degrees of nuclear labelling representing distinct active states of the NFκB system [45]. It is also important to note that neurons with low or high levels of NFκB activity and neurons considered to be inactive were adjacent to each other in the myenteric ganglia, reflecting the cellular heterogeneity of NFκB activation [45].

In order to achieve a more accurate view about the subcellular localisation of NFκB, immunogold electron microscopy was applied in the myenteric ganglia and neighbouring smooth muscle cells of the small intestine. Confirming the results of fluorescent and ELISA studies, in the ileum of diabetic rats, the total density of NFκB p65-labelling gold particles increased in both the ganglia and smooth muscle. Furthermore, this increase was manifested in both the nuclei and the cytoplasm of cells. The nuclear increase in the NFκB p65 subunit suggests its translocation leading to regulate the long-term expression of numerous target genes involved in inflammation and immunity [29,48]. According to our recent findings, both the expression of IL1β inflammatory cytokine and Toll-like receptor 4-sensing microbial lipopolysaccharides were markedly induced in myenteric neurons of this particular gut segment of type 1 diabetic rats [35,38]. IL1β and bacterial lipopolysaccharides are among the main triggers of the NFκB canonical pathway [29]; therefore, we propose that they largely contribute to the increased expression and activation of NFκB in the ileum of diabetic animals.

However, in the duodenum of diabetics, the total density of NFκB p65 gold labels remained unchanged in the ganglia and increased in the smooth muscle (both nuclear and cytoplasmic level), which supports findings that an increased tissue concentration of NFκB p65 in muscle/myenteric plexus homogenates derives from the intestinal smooth muscle in this segment. Moreover, the invariable NFκB p65 density in neuronal nuclei indicates again the lack of NFκB nuclear translocation and activation in the diabetic duodenum despite enhanced cytokine expression here [34,35]. It is known that the transport of NFκB p65 from the cytoplasm to the nucleus is mediated by importin α3 [49]; therefore, further detailed investigation of the importin transportation system is aimed in our future plans.

To reveal the underlying processes that led to our NFκB findings in the duodenum, we evaluated the diabetes-related alterations in Nrf2 expression, focusing primarily on this intestinal region. According to our electron microscopic findings, the total density of Nrf2-labelling gold particles increased in the ganglia and adjacent smooth muscle cells. It should be emphasized that enhanced Nrf2 expression was accompanied by a nuclear Nrf2 increase in the myenteric neurons. The nuclear translocation of Nrf2 leads to the transcription of different antioxidants, which are expressed at higher levels in the duodenum than in other gut segments of diabetic rats [50]. Other than the upregulation of the antioxidant defence system, Nrf2 is able to downregulate the NFκB signalling and protects cells in the gastrointestinal tract against oxidative stress [32]. In a diabetic mouse model, Nrf2 overexpression also inhibited the DNA-binding activity of NFκB [51]. Additional studies described that the stimulation of Nrf2 signalling decreases oxidative damage and inflammation and alleviates diabetic neuropathy [52,53].

## 5. Conclusions

Similar to the regional cytokine induction in diabetic rats [34,35], the NFκB expression has also shown a strict regionality according to intestinal segments, which suggests that the different cytokines also trigger the activation of the NFκB signalling pathway in the myenteric ganglia. However, non-uniform alterations were observed in the ganglionic NFκB expression of different gut segments in which the neuronal environment (e.g., intestinal redox status, antioxidant defence) has to play a critical importance [7]. Earlier, we demonstrated that myenteric neurons located in different gut segments displayed different susceptibilities to diabetic damage, and, according to this study, the duodenum was the only segment in which diabetic cell loss was not observed [6]. Therefore, we presume that the activation of Nrf2 in myenteric neurons may inhibit the NFκB pathway in the ganglia of the duodenum and may contribute to neuronal survival through the enhancement of antioxidant mechanisms in this particular intestinal segment in type 1 diabetes [40,54]. However, considering the complexity of NFκB-Nrf crosstalk and NFκB signalling, further investigations of the pathway components are required.

## Figures and Tables

**Table 1:** Primer sequences with accession number.

Gene	Primers (5′ → 3′)
NFκB (NM_199267)	CCGGAACTCTGGGAGCTGCC AGGCTAGGGTCAGCGTATGG
NRF2 (XM_006234397)	GCAACTCCAGAAGGAACAGG GGAATGTCTCTGCCAAAAGC

NFκB—nuclear factor kappa-light-chain-enhancer of activated B cells, NRF2—nuclear factor erythroid 2-related factor 2.

**Table 2:** Weight and blood glucose characteristics of the experimental animals.

	Body Weight (g)		Blood Glucose Concentration (mmol/L)	
	Initial	Final	Initial	Final (Average)
Controls (*n* = 14)	256.7 ± 17.68	467 ± 16.13 ****	5.96 ± 0.26	5.92 ± 0.11
Diabetics (*n* = 14)	223.9 ± 7.54	320.9 ± 14.73	6.09 ± 0.37	29.46 ± 0.87 ****^,oooo^
Insulin-treated diabetics (*n* = 13)	233.7 ± 9.44	463.1 ± 14.87 ****	5.86 ± 0.35	12.98 ± 1.5 *^,o^

Statistics: Kruskal–Wallis test with a Dunn’s multiple comparisons test. Data are expressed as mean ± SEM; * *p* < 0.05, **** *p* < 0.0001 vs. initial; ^o^ *p* < 0.05, ^oooo^ *p* < 0.0001 vs. controls.

**Table 3:** Intracellular distribution of Nrf2-labelling gold particles in myenteric neurons and intestinal smooth muscle cells (labels/µm^2^).

	Myenteric Neurons		Smooth Muscle Cells	
	Perikarya	Nuclei	Cytoplasm	Nuclei
Controls	1.12 ± 0.16	0.95 ± 0.06	0.67 ± 0.04	1.27 ± 0.11
Diabetics	1.43 ± 0.09 **	1.28 ± 0.08 *	0.92 ± 0.05 ***	1.35 ± 0.10
Insulin-treated diabetics	1.32 ± 0.09 *	1.16 ± 0.07	0.75 ± 0.04 °	0.97 ± 0.08 °

Statistics: Kruskal–Wallis test with a Dunn’s multiple comparisons test. Data are expressed as mean ± SEM; * *p* < 0.05, ** *p* < 0.01, *** *p* < 0.001 vs. controls; ° *p* < 0.05 vs. diabetics.

## Data Availability

Dataset available from the corresponding author at bodi.nikolett@bio.u-szeged.hu e-mail address.
